# North American survey and systematic review on caudal Septoplasty

**DOI:** 10.1186/s40463-020-00435-4

**Published:** 2020-06-08

**Authors:** Béatrice Voizard, Mélanie Theriault, Selma Lazizi, Sami P. Moubayed

**Affiliations:** Department of Surgery, Division of Otolaryngology – Head and Neck Surgery, Université de Montréal, 5400 Gouin Ouest Montreal, Quebec, Canada

**Keywords:** Nasal obstruction, Patient reported outcome measures, Caudal septal deviation, Anterior septal deviation, Septoplasty, Septorhinoplasty

## Abstract

**Background:**

Surgical correction of caudal septal deviation is a technically challenging step of functional rhinoplasty. Multiple surgical techniques have been described in the literature but comparing the efficacy of each in relieving obstruction presents a challenge. Outcome measures are necessary to adequately compare techniques. This study aims to describe the current caudal septoplasty techniques of Otolaryngologists and Facial plastic and reconstructive surgeons (FPRS), as well as their use of outcome measures, and to compare these practices with surgical trends described in the literature.

**Methods:**

An online survey was sent to three Otolaryngology and FPRS associations in Canada and the United States. A systematic review was conducted on SCOPUS and PubMed to classify the caudal septoplasty techniques described in the literature and the outcome measurement tools used.

**Results:**

Our survey identified that caudal septoplasty is more commonly performed by surgeons with an FPRS training background. The most common techniques were the swinging door technique (69.5%), extracorporeal septoplasy (46.7%), cartilage scoring (45.3%), and splinting with bone (25.4%). Despite using a vast array of surgical techniques, North American physicians rarely rely on standardized outcome assessment tools. Patient reported outcome measures (PROMs) are used almost twice as frequently in the literature as they are by surgeons in their clinical practice.

**Conclusion:**

We recommend that future studies of caudal septoplasty include an assessment of both form and function using a validated PROM such as the Standardized Cosmesis and Health Nasal Outcomes Survey.

## Background

**Fig. 1:**
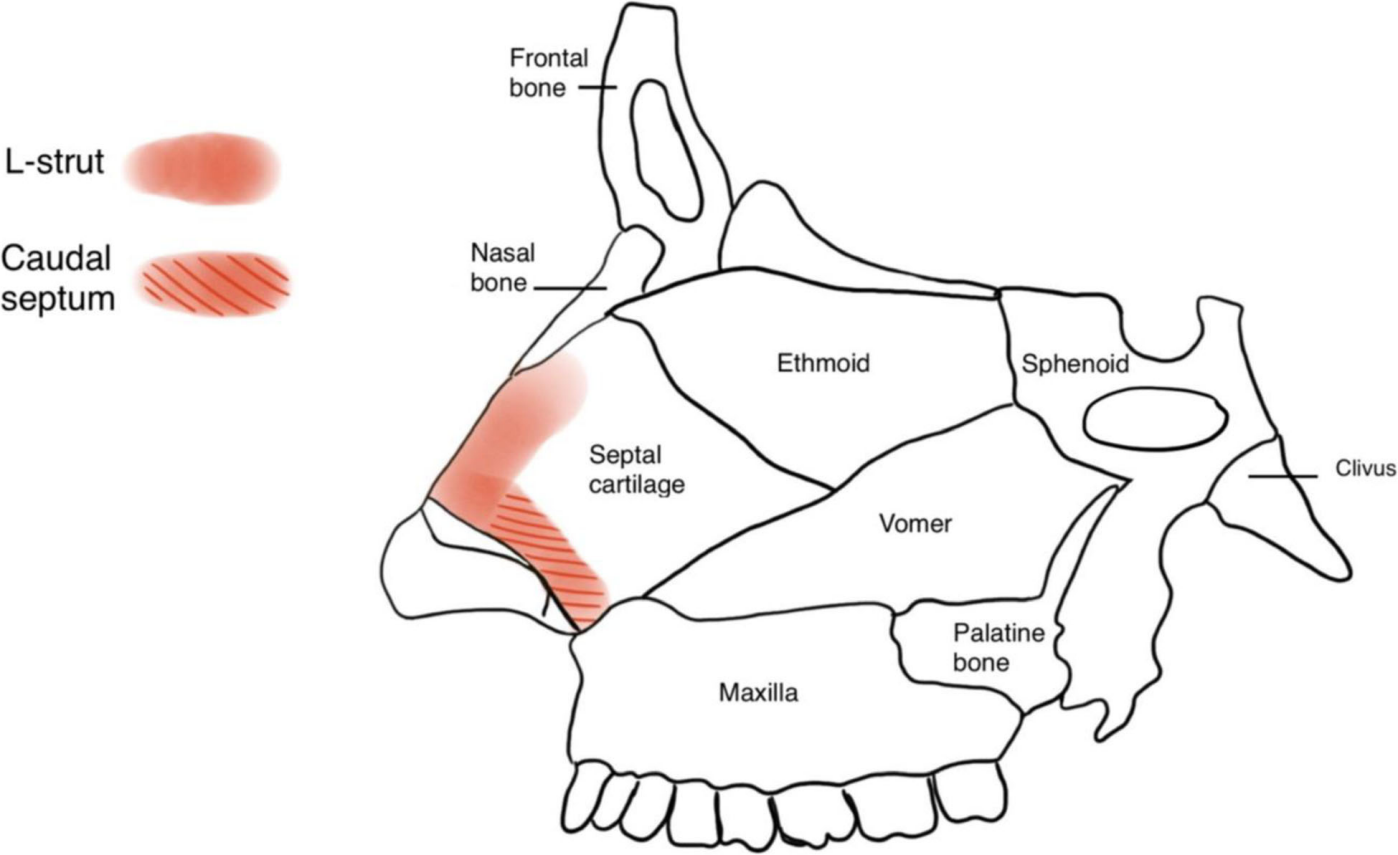
Anatomy of the caudal septum

Caudal septal deviation is defined as deviation of the anterior most portion of the nasal septum. (Fig. 1) In addition to functional symptoms, caudal septal deviation can cause significant cosmetic deformities, including lobule deviation, tip ptosis, and deformity of the middle one-third of the nose [1, 2]. Caudal septal deviation differs from traditional septal deviation in that it involves a portion of the septum that contributes to the nasal valve area and the support of the nasal tip [1].

Consequently, surgical correction of caudal septal deviation is technically challenging and classifies as a functional rhinoplasty procedure because of its impact on the external nasal valve. Removal of the deviated caudal nasal septum results in loss of tip support, shortening of the columella and complications ranging from tip ptosis [1] to severe valve collapse. Inadequate surgical correction can cause persistence of nasal obstruction postoperatively [3].

Multiple different surgical techniques have been described in the literature but comparing the efficacy of each in relieving functional symptoms presents a challenge. Objective parameters measured by acoustic rhinometry (AR) and rhinomanometry (RM) do not correlate with patients’ perceived sensation of obstruction [4]. Visual analog scales (VAS) and patient-reported outcome measures (PROMs) were conceived to collect standardized assessments of each patient’s subjective symptoms and overall satisfaction in the preoperative and postoperative periods [5]. The Standardized Cosmesis and Health Nasal Outcomes Survey (SCHNOS) is a PROM that meets international methodological guidelines that has been validated specifically for the evaluation of cosmetic and functional outcomes after septorhinoplasty. The use of PROMs, VAS, AR and RM to report functional outcomes after caudal septoplasty varies widely in the literature.

The primary objective of this study was to describe the current caudal septoplasty techniques of otolaryngologists and facial plastic surgeons, as well as their use of outcome measures.

The secondary objective was to compare these practices with the surgical techniques and outcome measures described in the literature, by conducting a systematic review.

## Methods

Scientific and ethics committee approval to undergo this project was obtained at our institution (Hopital Maisonneuve Rosemont), followed by approval from each surveyed medical association. An English or French, 9-question survey requiring on average 1,5 min to complete was sent via email. The survey was designed using web survey development cloud base SurveyMonkey (www.surveymonkey.com, SurveyMonkey, Palo Alto, CA). The survey was distributed via email to members of the following associations; the Association of Otolaryngology-Head and Neck Surgery of Quebec (ORLQC), the Canadian Society of Otolaryngology–Head and Neck Surgery (CSOHNS) and the American Association of Facial Plastic and Reconstructive Surgery (AAFPRS).

Each association received a cover letter describing the purpose of the survey and containing a link to the survey. Participants did not receive any compensation. The survey links were kept active for 3 months after the last email was sent out to the members by their association. The respondents were prevented from responding more than once by the online platform SurveyMonkey.

The survey included questions regarding demographic characteristics such as current practice location, country in which residency was completed, completion of a fellowship in facial plastic and reconstructive surgery, exposition to extracorporeal septoplasty during training, type of practice (academic, community, other), years of practice and number of patients with caudal septal deviation seen per month. Respondents were then asked to state how they evaluate nasal obstruction (history, physical examination, standardized questionnaire, airflow measurements, other) and the technique they use to treat caudal septal deviation (scoring, swinging door, splinting with bone, extracorporeal septoplasty, other). Respondents were allowed to select multiple answers to this question, since many techniques include combinations.

We then conducted a systematic literature review following the PRISMA statement guidelines [6, 7]. The two databases used were PubMed and SCOPUS. The keywords used were [“caudal deviation” OR “caudal septal deviation” OR “anterior deviation” OR “anterior septal deviation”]. No limitations were added. All studies published in French or English between 1954 and 2018 were included.

All included studies met the following criteria: articles including a description of a surgical technique (either septoplasty or septorhinoplasty) aiming to correct caudal septal deviation; procedures addressing primarily functional symptoms; articles providing enough detail to understand the steps of the procedure and to differentiate it from other techniques; articles detailing how functional outcomes were evaluated; articles stating the number of patients on which the outcomes were measured, and stating the number of controls, if controls were used.

The exclusion criteria were the following: articles not in French or English, procedures addressing non-caudal nasal septal deviations; articles evaluating only or primarily cosmetic outcomes. Literature reviews, correspondences, descriptions of techniques without any outcome evaluation, and book chapters or sections were excluded from this review.

**Fig. 2:**
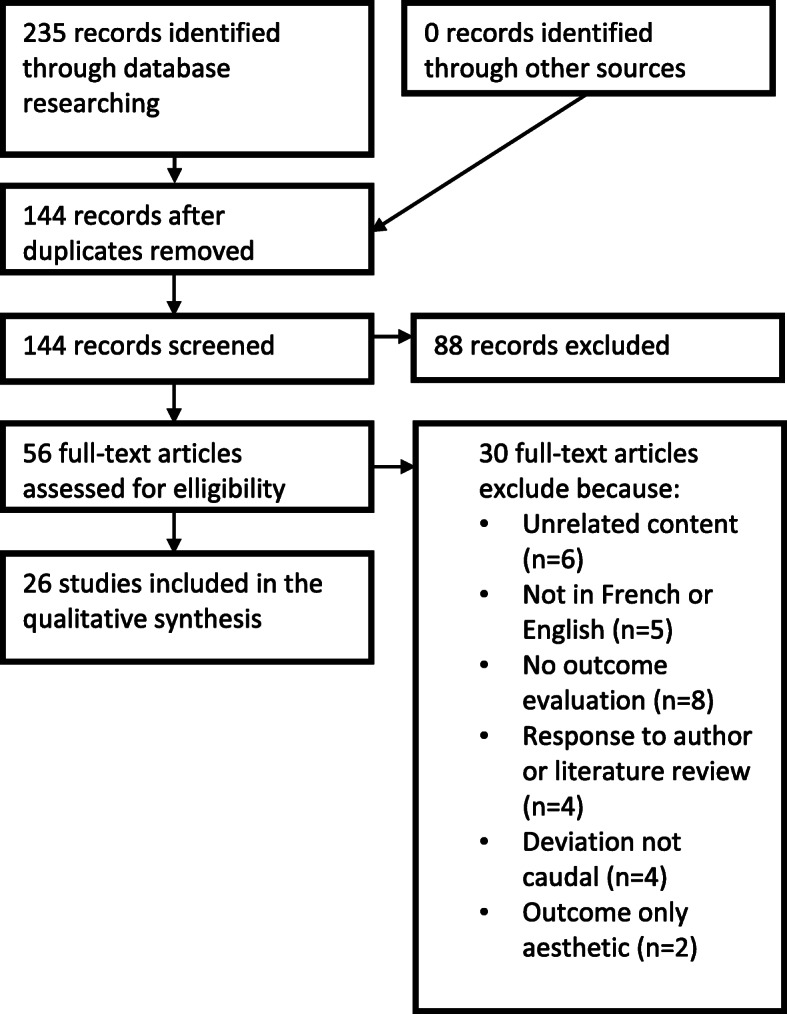
Literature Review Flow Diagram

After identifying records through database researching and removing duplicates, abstracts were screened for eligibility by two independent readers and classified as “included”, “excluded” or “neither excluded or included”. A tertiary, independent reader further classified the “neither excluded or included” abstracts, deciding whether they would be included or not. Included full-text articles were read and analyzed, and further articles were excluded. A qualitative synthesis is presented in this article. Figure 2 is a flow diagram representing the number of records identified, included and excluded, and reason for exclusion when applicable.

## Results

### Survey

**Fig. 3:**
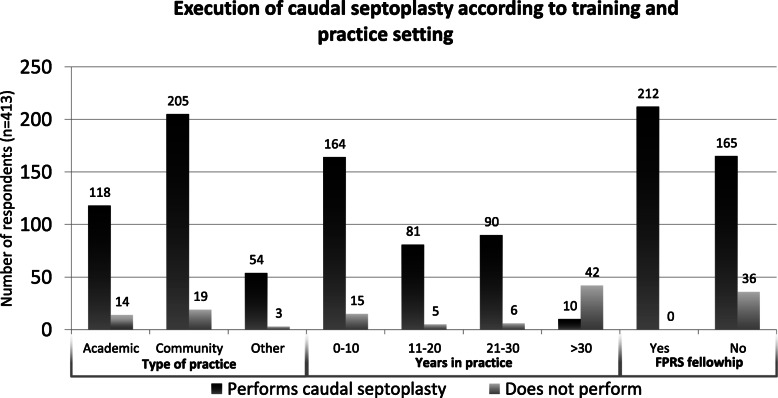
Execution of caudal septoplasty according to training and practice setting

Table 1 regroups the demographic characteristics of all respondents. Out of 413 respondents, 87 were from ORLQC, 108 were from CSOHNS and 218 were from AAFPRS. This represents an estimated response rate of 36.9, 19.5 and 9%, respectively. The vast majority of respondents performed caudal septoplasty (*n* = 3277; 91.3%), and half (51.3%) of the respondents completed a facial plastic and reconstructive surgery (FPRS) fellowship. All respondents who completed an FPRS fellowship performed caudal septoplasty (Fig. 3). Respondents came either from academic (32%), community (54.2%) or other practice settings (13.8%). A high proportion of respondents were in the beginning of their career (< 10 years; 43.3%), but the proportion of respondents who performed septoplasty was similar among all practitioners with less than 30 years of practice (1st-10th: 91.6%; 11th–20th year: 94.2%; 21st-30th: 93.8%).

**Table 1:** Demographic characteristics of respondents

	Number of respondents	Percentage of respondents
Society of origin		
ORL QC	87	21.1%
CSOHNS	108	26.2%
AAFPRS	218	52.8%
Training		
Canada	193	46.7%
US	210	50.8%
Other	10	2.5%
Performing caudal septoplasty		
Yes	377	91.3%
No	36	8.7%
FPRS fellowship completion		
Yes	212	51.3%
No	201	48.7%
Practice setting		
Academic	132	32%
Community	224	54.2%
Other	57	13.8%
Years in practice		
1–10	179	43.3%
11–20	86	20.8%
21–30	96	23.2%
> 30	52	12.6%
Number of caudal deviations seen per month		
0–2	108	26.2%
3–5	140	33.9%
6–15	140	33.9%
> 15	25	6.1%

**Fig. 4:**
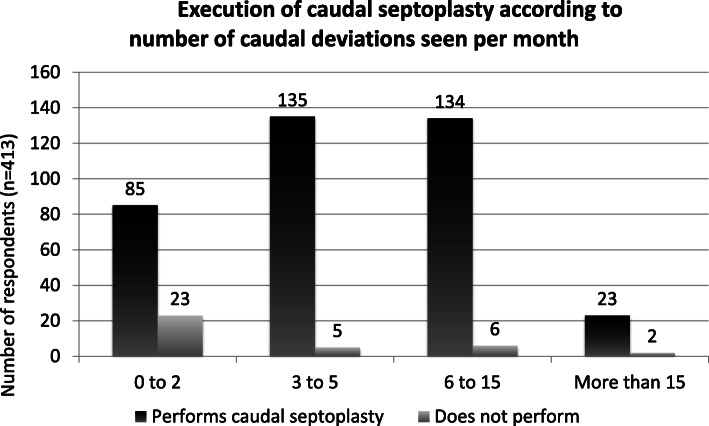
Execution of caudal septoplasty according to number of caudal deviations seen per month

Of the respondents who saw the least caudal septal deviations in clinic (0–2 cases/month), 21.3% did not perform caudal septoplasty. Among respondents seeing 3 to 15 cases per month, less than 5% did not perform caudal septoplasty. (Fig. 4).

**Fig. 5:**
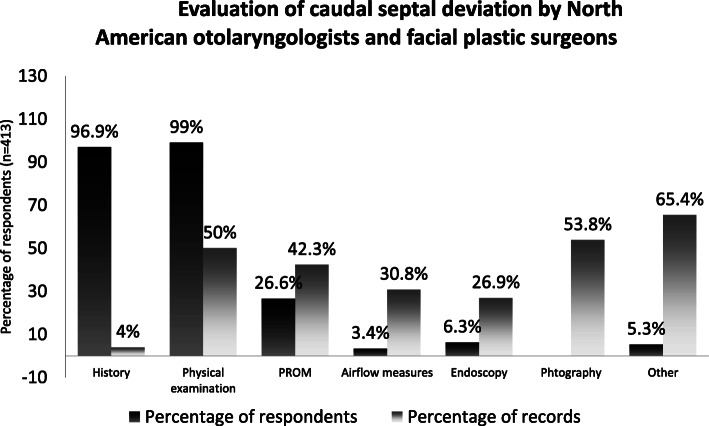
Evaluation of caudal septal deviation by North American otolaryngologists and facial plastic surgeons

A vast majority of respondents use history and physical examination (96.9, and 99%, respectively) to evaluate the degree of nasal obstruction. Only 26.6% of respondents use PROMs in their clinical practice. (Fig. 5).

**Fig. 6:**
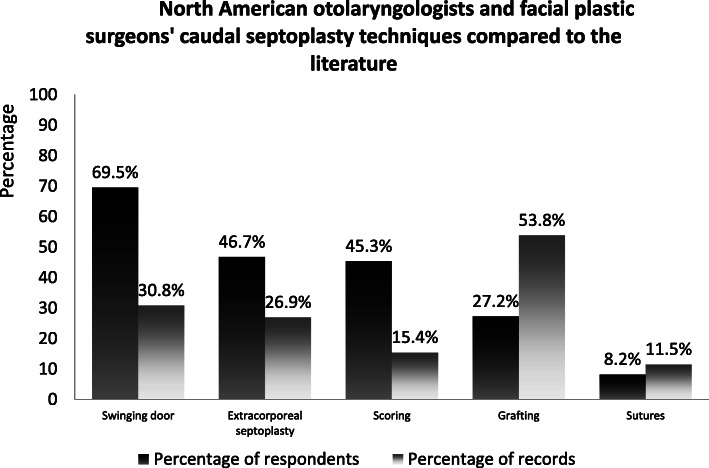
North American otolaryngologists and facial plastic surgeons’ caudal septoplasty techniques compared to the literature

The most popular surgical techniques were the swinging door technique (69.5%), extracorporeal septoplasty (46.7%), cartilage scoring (45.3%), and splinting with bone (25.4%). Other techniques were rarely employed (Fig. 6).

Surgeons who have completed an FPRS fellowship perform more caudal septoplasty than their counterparts (82% vs 100%, *p* < 0.001). Surgeons with an FPRS background utilize more often the swinging door (*p* < 0.001), splinting (*p* < 0.001), extracorporeal septoplasty (*p* < 0.001), and PDS plates (*p* < 0.001) than their counterparts. There was no difference in suture technique use between surgeons with or without an FPRS training background.

### Literature review

The initial database search yielded a selection of 235 records, of which 26 were included in the qualitative synthesis after application of exclusion criteria (Fig. 2). These studies were published between 1994 and 2017. The number of patients included varied from 2 [8] to 703 [9] with a total of *n* = 1880 cases (Table 2). Only four articles [12, 19, 20, 22] (15%) included a control group.

**Table 2:** Literature Review

Author, year	Surgical Technique	Number of cases	Outcome evaluation methods
Aboul Wafa. 2017 [10]	Swinging Door; Grafting	18	CQ; PA
Akduman, D., et al. 2014 [11]	Swinging Door	36	PA
Calderon-Cuellar, L. T., et al. 2004 [12]	Suturing; Scoring	25	CE; RM; CQ
Chung, Y. S., et al. 2014 [13]	Swinging Door; Grafting	39	CE; E; AR; VAS; CQ
Constantine, F. C., et al. 2014 [8]	Swinging Door; Grafting	2	PA
Dyer, W. K. et al. 2000 [14]	Swinging Door; Grafting	36	CE; CQ; PA
Garcia, L. B., et al. 2001	Grafting	10	CE; AR; NOSE; PA
Giacomini, P., et al. 2010 [15]	Grafting; Scoring	15	CE; RM; NOSE; PA
Indeyeva, Y. A., et al. 2017 [16]	Suturing; Other	148	HPI
Jang, Y. J., et al. 2009 [17]	Grafting	45	E; VAS
Kamami, Y. V. 1997 [18]	Other	120	CE; E; AR; CQ
Kamami, Y. V., et al. 2000 [9]	Other	703	CE; E; AR; CQ
Karadavut, Y., et al. 2017 [19]	Grafting	20	CE; AR; NOSE; ROE
Kayabasoglu, G., et al. 2015 [20]	Extracorporeal	45	CE; NOSE; PA
Kim, D. Y., et al. 2017 [21]	Grafting	141	E; NOSE; CQ
Kim, J. H., et al. 2011 [22]	Grafting; Scoring	56	VAS; CQ
Koch, C. A., et al. 2011 [23]	Extracorporeal; Grafting	10	CQ; PA; Other
Lee, J. W., et al. 2013 [3]	Grafting	66	CE; CQ; PA
Loyo, M., et al. 2017 [24]	Extracorporeal	71	NOSE; PA
Metzinger, S. E., et al. 1994 [25]	Swinging Door; Grafting	10	CE; CQ; PA
Most, S. P. 2006 [26]	Extracorporeal; Grafting	12	NOSE; PA
Murrell, G. et al. 2000 [27]	Extracorporeal	10	CE; PA
Sedwick, J. D., et al. 2005 [28]	Swinging Door	62	PA
Shin, J. H., et al. 2011 [29]	Swinging Door; Suturing	40	AR; VAS
Surowitz, J., et al. 2015 [30]	Extracorporeal	77	NOSE; VAS
Yaniv, D., et al. 2017 [31]	Extracorporeal; Scoring	63	CE; E; ROE; SNOT-16

*CQ* Custom questionnaire, *PA* Photographic analysis, *CE* Clinical examination, *E* Endoscopy, *HPI* History of present illness, *RM* Rhinomanometry, *AR* Acoustic rhinometry, *VAS* Visual assessment scale, *NOSE* Nasal Obstruction Symptom Evaluation, *ROE*: rhinoplasty outcome evaluation, *SNOT-16* SinoNasal Outcome Test-16

Studies were classified by the surgical technique described. Since some records described a combination or sequence of surgical techniques, these records have been included simultaneously in multiple categories, as for the survey. To prevent classification errors, categories were precisely described before classifying each record, as follows: techniques that moved the lowermost part of the caudal septum to the midline or contralateral to the anterior nasal spine were categorized as a “swinging door technique”. The “extracorporeal septoplasty” technique was defined as the removal of deviated cartilage with or without bony septum, followed by extracorporeal remodeling and subsequent reimplantation. The “splinting or grafting technique” includes cartilage splinting, and various bony or cartilaginous grafts used to stabilize the nasal septum. Cartilage grafts consisted either of posterior septal, quadrangular, costal or conchal cartilage. Bone grafts all consisted of bony batten grafts of various origins. “Suturing” regrouped all techniques consisting of permanent trans-cartilaginous retention sutures. “Scoring” techniques comprised all techniques where partial thickness incisions were made in the cartilage to overcome its natural bending forces.

Included records were also classified according to primary surgical outcome. Outcome was only functional for 15 records [9–18, 20–22, 29, 30] (58%), while 11 [3, 8, 19, 23–28, 31, 32] (42%) additionally evaluated esthetical outcomes.

**Fig. 7:**
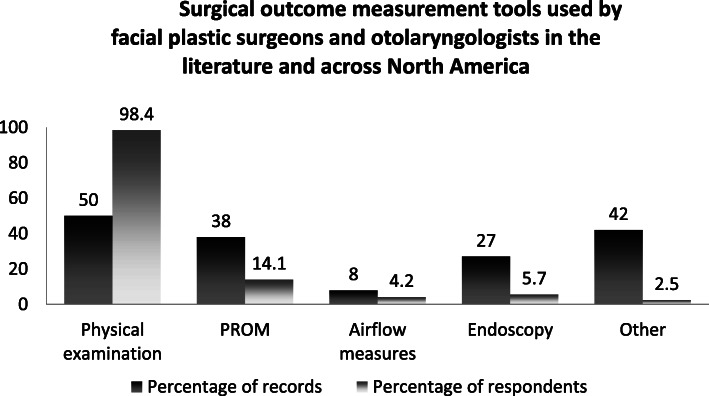
Surgical outcome measurement tools used by facial plastic surgeons and otolaryngologists in the literature and across North America

As cited in 50% of the records, physical examination is the most popular way to evaluate post-operative relief of nasal obstruction. History taking was not explicitly cited in many articles, but the use of endoscopy (26.9%), and airflow measure such as RM (7.7%) or AR (23.1%) were described (Fig. 7).

Administration of questionnaires was common (42.3%), and the NOSE (30.8%) was the most commonly used. The average results of the 8 studies [15, 19–21, 24, 26, 30, 33] that used the NOSE questionnaire were compared. The average calculated NOSE score was 19.3 post-operatively compared to 72.0 preoperatively for all techniques combined (Table 3). Aside from the NOSE, other PROMs such as the rhinoplasty outcome evaluation (ROE) [19, 31] (7.7%), and the 16-item Sino-Nasal Outcome test (SNOT-16, 27) (3.8%) were also reported. Custom questionnaires (42.3%) were often used. Out of the 11 records using custom questionnaires [3, 9, 10, 12–14, 18, 21–23, 25], only 3 [13, 21, 25] disclosed the questions and rating methods fully.

**Table 3:** Average preoperative and post-operative NOSE scores

Author	Surgical Technique	Number of cases	Preoperative NOSE score	Postoperative NOSE score
Garcia, L. B., et al.	Grafting	10	82.7	7.4
Giacomini, P., et al. [15]	Grafting; Scoring	15	57.4*	23.7^a^
Karadavut, Y., et al. [19]	Grafting	20	64.0	12.0
Kayabasoglu, G., et al. [20]	Extracorporeal	45	85.0*	25.0^a^
Kim, D. Y., et al. [21]	Grafting	141	70.5	28.7
Loyo, M., et al. [24]	Extracorporeal	71	72.3	24.0
Most, S. P [26].	Extracorporeal; Grafting	12	76.6	12.9
Surowitz, J., et al. [30]	Extracorporeal	77	68.2	21.1

^a^NOSE scores had to be recalculated because they were not reported according to standard NOSE score calculation within each article

As previously mentioned, 11 [3, 8, 19, 23–28, 31, 32] (42.3%) records evaluated cosmetic outcomes in addition to functional outcomes. Out of all the records, 53.8% reported a post-operative photographic analysis of the nose. Of these, 21.4% [8, 11, 28] used photography as the only tool to quantify post-operative improvement, for both functional and esthetic outcomes. None used the SCHNOS.

## Discussion

To our knowledge, this is the first survey in the literature to evaluate the use of caudal septoplasty techniques and outcome measurement tools. The aim of this descriptive study was to compare the different surgical techniques described in the literature to those used by surgeons today, especially with regards to the use of patient-reported outcome measures. As for any descriptive study, the objective is to portray current surgical trends rather than to draw conclusions regarding the efficacy of one technique over others.

Classifying caudal septoplasty techniques presents a challenge, due to the vast spectrum of minor surgical variations described in the literature to this day. As shown in Table 2, of the 26 records included in this systematic literature survey, only two [9, 18] could not be likened to one of the five most commonly used techniques. Other ones were variants of existing techniques. Although this regrouping relies on the reviewer’s comprehension, which inevitably induces a bias, comparisons can be made. Figure 6 highlights that swinging door is more popular amongst survey respondents, whereas that grafting and splinting techniques were described more often in the literature. This could be attributable to a publication bias, since surgeons describing their techniques are more likely to be experts in their field, who rely on more complex surgical techniques in secondary or tertiary reference centers.

### Comparing the literature to survey answers

A dual rationale underlies the comparison of survey answers and literature review results. First, it highlights a disparity between the techniques commonly being used by North-American otolaryngologists and facial plastic surgeons compared to what the literature depicts, which is presumably what is being done by experts in the field. Essentially, a large toolbox exists, but surgeons in practice - 54% of which were community-based - may be focusing on mainly one technique, (swinging door, see Fig. 6). Why are surgeons preconizing technique when so many have been described? Reliable outcome data could allow the otolaryngologist or facial plastic surgeon to make an informed clinical choice. This leads to the second purpose of the comparison. It shines light on the fact that valid standardized tools such as PROMs aren’t systematically used, either in practice *nor* in the literature. No one would do a stapedotomy without a preoperative audiogram, but surgeons operate on the deviated caudal septum without using standardised outcome assessment tools, *both* in clinical practice and in the literature.

### Comparing surgical outcomes

Measuring performance in rhinoplasty can be a challenge, although previously published guidelines [34] recommend the utilization of PROMs at least 1 year after surgery. In the present systematic review, only 8 records used the NOSE score to compare preoperative and post-operative obstruction. Because of the relatively low number of patients and high heterogeneity between records, no valid meta-analysis has been possible.

Surprisingly, PROMs are not very prevalent in routine clinical practice, possibly due to logistic reasons. Moreover, the present study highlights that PROMs are used almost twice as frequently in the literature as they are by surgeons in their clinical practice (Fig. 5).

In October 2018, the American Society of Plastic Surgeons, in a joint effort with the AAFPRS and the AAO-HNS released a set of performance measures for rhinoplasty and recommended that the SCHNOS questionnaire be administered at least preoperatively and 1 year postoperatively to rhinoplasty patients as a performance measure [34].

Comparing preoperative and postoperative PROM scores between one technique and another could provide with a simple surgical efficacy ranking. However, functional rhinoplasty is an operation where both cosmesis and function must be measured. In our systematic review, no study evaluated both form and function after caudal septoplasty using PROMs, rendering the evaluation of performance difficult. We recommend that future studies evaluating functional rhinoplasty techniques include both a pre- and postoperative evaluation of both form and function using a PROM that can evaluate both, such as the SCHNOS.

In the present study, we excluded records that did not report surgical outcomes. This criterion yielded a selection of mostly recent articles, published after 2010. Many older records were excluded for a lack of outcome evaluation. In the present literature review, custom questionnaires were used more commonly then PROMs.

## Conclusion

In conclusion, our survey identified that caudal septoplasty is more commonly performed by surgeons with an FPRS training background. The most common techniques were the swinging door technique (69.5%), extracorporeal septoplasy (46.7%), cartilage scoring (45.3%), and splinting with bone (25.4%). Our systematic review identified no studies that evaluated both functional and cosmetic outcomes of any technique with validated outcome measurement tools. We recommend that future studies of caudal septoplasty include an assessment of both form and function using a validated PROM such as the SCHNOS, as recommended by the American Society of Plastic Surgeons.

## Data Availability

The datasets used and analyzed during the current study are available from the corresponding author on reasonable request.
